# Three-column osteotomy surgery versus standard surgical management for the correction of adult spinal deformity: a cohort study

**DOI:** 10.1186/s13018-015-0154-3

**Published:** 2015-02-03

**Authors:** Xinran Ji, Hua Chen, Yiling Zhang, Lihai Zhang, Wei Zhang, Sigurd Berven, Peifu Tang

**Affiliations:** Department of Orthopaedic Surgery, The General Hospital of People’s Liberation Army (301 Hospital), 28 Fuxing Road, Wukesong, Beijing, 100000 China; Department of Orthopaedic Surgery, Spine Fellowship and Resident Education Program, University of California, San Francisco, 500 Parnassus Ave., MU320W, San Francisco, CA 94143-0728 USA; Department of Orthopaedic Surgery, Medical Center, University of California, San Francisco, 500 Parnassus Ave., MU320W, San Francisco, CA 94143-0728 USA

**Keywords:** Standard surgical management, Three-column osteotomy, Adult spinal deformity

## Abstract

**Background:**

The aim of this study was to analyze and compare the surgical data, clinical outcomes, and complications between three-column osteotomy (3-COS) and standard surgical management (SSM) for the treatment of adult spine deformity (ASD).

**Methods:**

A total of 112 patients who underwent consecutive 3-COS (*n* = 48) and SSM (*n* = 64) procedures for ASD correction at a single institution from 2001 to 2011 were reviewed in this study. The outcomes were assessed using the Scoliosis Research Society (SRS)-22 scores. The complications of patients with 3-COS and SSM were also compared.

**Results:**

No significant differences were found in patient characteristics between SSM and 3-COS groups. Surgical data and radiographic parameters showed that the patients of the 3-COS group suffered more severe ASD than those of the SSM group. The distribution of surgical complications revealed that SSM group underwent more complications than 3-COS groups with no significant differences. At final follow-up, the total SRS-22 score of SSM was not significant between pre-operation and post-operation. However, the total SRS-22 score of 3-COS at final follow-up was significantly higher than pre-operation.

**Conclusion:**

For severe ASD patients with high grade pelvic incidence (PI), pelvic tilt (PT), and PI/lumbar lordosis (LL) mismatch and who have subjected to spine surgeries more than twice before, 3-COS might be more effective than SSM in improving the clinical outcomes. However, due to the higher reoperation rate of 3-COS, SSM may be more appropriate than SSM for correcting the not serious ASD patients.

## Introduction

Adult spinal deformity (ASD) is defined as a complex spectrum of spinal conditions presented in adults such as degenerative scoliosis, kyphosis, and iatrogenic deformity [1]. Surgical procedures for ASD, including standard surgical management (SSM) and three-column osteotomy (3-COS), have gained popularity over the last decade [2]. As the correction of spinal deformity is difficult, the goals of ASD surgery are to alleviate pain, stop deformity progression, and improve function. SSM, including anterior, posterior, or lateral approach inter body fusion surgery, had been proved to be effective in improving the radiographic and functional outcomes for ASD [1]. However, if the ASD patients with severe and rigid curves want to achieve adequate correction of the deformity, a 3-COS surgery is required.

The aim of the 3-COS is to correct and provide a balanced spine with reasonable amount of correction. There are three osteotomies for correcting deformities, including Smith-Petersen osteotomy (SPO), pedicle subtraction osteotomy (PSO), and vertebral column resection (VCR) [1,2]. SPO is a posterior column osteotomy which removes the posterior ligaments and facet joints, and mobile anterior disc is required for correction. PSO is a technique of removing all of the posterior parts, both the pedicle and half of the vertebral body. It is usually performed for the treatment of idiopathic or iatrogenic flatback deformity with fixed sagittal imbalance (FSI). VCR involves complete resection of one or more vertebral segments including posterior elements, entire vertebral body, and adjacent discs and enables significant deformity correction in all three dimensions [2,3]. Recently, VCR began to be used as a technique for the treatment of severe and rigid spinal deformities [4-7].

Spine surgery for ASD patients is expected to be the final therapeutic intervention in management. Low reoperation rates are ideal, but complications or other problems could increase the risk of reoperation [8-12]. Regardless of corrective results, ASD surgeries always bring some complications. Some published studies have reported that the complication rate is more than 40% [6,13-16]. Some complications such as postoperative kyphotic decompensation syndrome and proximal junctional kyphosis (PJK) are challenges of the spine surgery [4,17-20]. Complication incidences of the ASD patients after 3-COS and SSM have not been compared. In addition, limited studies have delved into the differences of complications and other prognostic factors between 3-COS and SSM in the treatment of ASD. The purpose of this study therefore was to evaluate the safety and efficacy of 3-COS compared with SSM for correcting ASD.

## Materials and methods

### Patient selection

This study was approved by the ethics committee of The General Hospital of People’s Liberation Army (301 Hospital). Written informed consents were obtained from all participants.

We included 112 patients who underwent consecutive 3-COS (*n* = 48) or SSM (*n* = 64) surgery at a single institution from 2001 to 2011 in this study. Preoperative diagnosis included idiopathic deformity (32 patients), degenerative deformity (31 patients), iatrogenic etiologies (41 patients), and other diagnoses (8 patients). All patients aged 21 or older at initial surgery, and they were treated by a multi-level (≥5 levels) spinal fusion and had a minimum 2 years follow-up. Patients with any major coronal, sagittal, or combined deformity requiring instrumented fusion were also included. However, patients with acute vertebral fracture, paraplegia, spinal tumor, active infection, and neuromuscular scoliosis were excluded. Radiographic and clinical data including SRS-22 score were used to assess the clinical outcomes.

### Data collection

The clinical data were reviewed to compare the following indexes or parameters: demographic characteristics, surgical characteristics, radiographic parameters, and complications. Demographic parameters including age, gender, weight, height, smoking history, body mass index (BMI, weight [kg]/height [m^2^]) were also collected.

Comorbidities such as diabetes, osteoporosis, hypertension, heart disease, respiratory disease, collagen disease, and liver disease were recorded. The SRS-22 questionnaire scores were used to evaluate preoperative outcomes and postoperative outcomes at the final follow-up of patients. SRS-22 includes five domains: function, pain, self-image, mental health, and satisfaction. Total scores were calculated and compared between two groups.

Radiographic parameters including sagittal vertical axis (SVA), lumbar lordosis (LL: L1 − S1), pelvic incidence (PI), pelvic tilt (PT), and sacral slope (SS) were obtained from standing long-cassette anteroposterior (AP) and lateral radiographs of ASD patients. Measurement techniques for spine-pelvic parameter, region curvatures, and sagittal alignment were carried out as Lafage et al. [12] described. According to the Schwab classification [21], we defined anterior global sagittal alignment as SVA ≥ 40 mm, lumbar hypo-lordosis as LL < 30°, high-grade PI as PI > 55°, high-grade PT as PT ≥ 20°, or PI/LL mismatch or PI minus LL ≥ 10°.

All complications including intraoperative events, perioperative events before discharge, or complications during the follow-up period were recorded.

### Statistical analysis

All data were presented as mean ± standard deviation. The unpaired *t*-test and chi-square test were used to compare the differences between-group. The paired *t*-test was used to compare changes in clinical outcomes between the preoperative and final follow-up evaluations. The log-rank test was used to compare the survival distributions of two values. *P* < 0.05 was considered statistically significant. Statistical analysis was performed using SPSS 16.0 (IBM Corp., Armonk, NY, USA).

## Results

### Patient characteristics

The characteristics for the entire cohort are shown in Table 1. There were no significant differences in any patient characteristics between SSM and 3-COS groups (*P* > 0.05).

**Table 1:** **Demographic data of the patients**

***N***	**SSM (** ***N*** **= 64)**	**3-COS (** ***N*** **= 48)**
Age at surgery (years)	52.3 ± 12.4	53.1 ± 12.7
Weight (kg)	70.3 ± 18.9	72.4 ± 18.7
Height (cm)	162.7 ± 8.9	161.1 ± 8.7
BMI (kg/m^2^)	27.3 ± 6.1	26.9 ± 5.8
Follow-up (months)	42.1 ± 17.2	47.1 ± 13.9
Hypertension	32 (50%)	15 (31.3%)
Respiratory disease	11 (17.2%)	8 (16.7%)
Osteoporosis	9 (14.1%)	10 (20.8%)
Heart disease	11 (17.2%)	9 (18.8%)
Diabetes mellitus	9 (14.1%)	7 (14.6%)
Smoking	9 (14.1%)	6 (12.5%)

*Abbreviations*: *BMI* body mass index, *SSM* standard surgical management, *3-COS* three-column osteotomy.

### Radiographic outcomes

As shown in Table 2, the surgical data and radiographic parameters showed that the patients of the 3-COS group suffered more severe ASD than those of the SSM group. Among these parameters, fused levels, SVA, PI, PT, and PI minus LL showed significant differences between the two groups (*P* < 0.05). In addition, the patients who have subjected more than twice of spine surgeries in the 3-COS group have significantly more effective clinical outcomes than those in the SSM group.

**Table 2:** **Clinical and radiographic characteristics of the patients**

	**SSM (** ***N*** **= 64)**	**3-COS (** ***N*** **= 48)**
Fused levels	9.1 ± 6.3	11.6 ± 4.2*
Fusion to sacrum	49 (76.6%)	38 (79.2%)
More than twice of spine surgeries before	24 (37.5%)	39 (81.3%)*
Preoperative SVA (mm)	55.2 ± 48.9	98.3 ± 80.9*
Preoperative LL (°)	31.5 ± 22.3	27.3 ± 25.1
Preoperative PI (°)	53.4 ± 11.3	58.7 ± 10.9*
Preoperative PT (°)	23.6 ± 10.7	28.1 ± 12.6*
Preoperative SS (°)	27.9 ± 10.5	29.3 ± 11.9
Preoperative PI minus LL (°)	21.8 ± 21.1	30.7 ± 20.8*

*Abbreviations: SSM* standard surgical management, *3-COS* three-column osteotomy, *SVA* sagittal vertical axis, *LL* lumbar lordosis, *PI* pelvic incidence, *PT* pelvic tilt, *SS* sacral slope.

*Statistically significant (*P* < 0.05).

**Figure 1:**
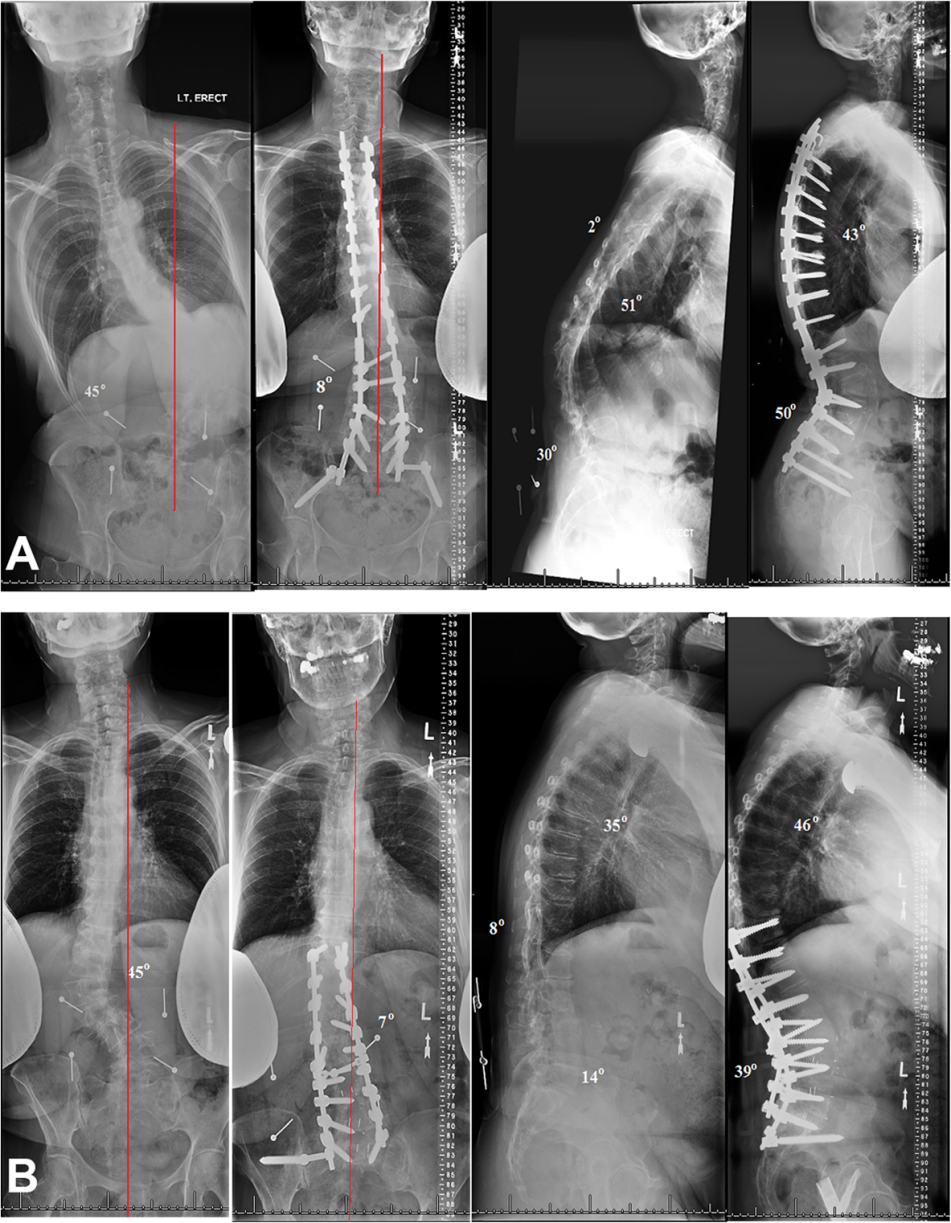
**Two 63- and 79-year-old women suffering from adult spinal deformity underwent 3-column osteotomy (A) and standard surgical management (B), respectively.** Three years after surgery, anteroposterior and lateral radiographs demonstrate marked correction, satisfactory alignment, and a solid spinal fusion.

As shown in Figure 1, preoperative and 3-year postoperative clinical photographs of two old women were displayed. The women who underwent 3-COS had more severe ASD than the one who underwent SSM. However, similar corrective results were observed. This result is in accordance with the outcomes of the cohort in our study.

### Surgical complications

The distribution of surgical complications was shown in Table 3. Overall, 18 (28.1%) patients of the SSM group underwent complications, which was more than that of 3-COS (11 of 48, 22.9%), but no significant differences were observed. In addition, there were no significant differences between SSM and 3-COS groups in any complication (*P* > 0.05).

**Table 3:** **Surgical complications**

	**SSM (** ***N*** **= 64)**	**3-COS (** ***N*** **= 48)**
Infection (*N* = 8)	5 (7.8%)	3 (6.3%)
Neurologic complications (*N* = 6)	4 (6.3%)	2 (4.2%)
Implant trouble (*N* = 3)	1 (1.6%)	2 (4.2%)
Pseudarthrosis (*N* = 5)	3 (4.7%)	2 (4.2%)
ASP and PJK (*N* = 7)	5 (7.8%)	2 (4.2%)
Total	18 (28.1%)	11 (22.9%)

*Abbreviations: SSM* standard surgical management, *3-COS* three-column osteotomy, *ASP* adjacent segment problem, *PJK* proximal junctional kyphosis.

### Clinical outcomes

As shown in Table 4, the total preoperative SRS-22 score of the 3-COS group was significantly lower than that of the SSM group. Also, the function, pain, and self-image scores of the 3-COS group at pre-operation were significantly lower than those of the SSM group (*P* < 0.05). At final follow-up, the total SRS-22 score was no more significant between SSM and 3-COS group. In addition, no significant change was observed in the total SRS-22 score in SSM group after treatment. However, the total SRS-22 score of the 3-COS group at final follow-up was significantly higher than the total score at pre-operation (*P* < 0.05). In addition, function and pain scores of subjects with SSM at final follow-up were significantly higher than those of 3-COS.

**Table 4:** **SRS-22 scores before and after treatment by 3-COS and SSM**

**SRS-22**	**Pre-operation**		**Post-operation** ^**a**^	
	**SSM**	**3-COS**	**SSM**	**3-COS**
Function	3.54 ± 0.61	3.10 ± 0.67*	3.61 ± 0.57	3.32 ± 0.38*
Pain	2.91 ± 0.14	2.53 ± 0.61*	3.42 ± 0.67**	2.98 ± 0.74*,**
Self-image	3.42 ± 0.54	3.14 ± 0.61*	3.64 ± 0.56**	3.51 ± 0.63**
Mental health	3.57 ± 0.64	3.49 ± 0.76	3.79 ± 0.73	3.81 ± 0.59**
Satisfaction	3.24 ± 0.71	3.17 ± 0.82	3.97 ± 0.53**	3.88 ± 0.65**
Total	3.32 ± 0.41	2.97 ± 0.81*	3.66 ± 0.63	3.51 ± 0.59**

*Abbreviations: SSM* standard surgical management, *3-COS* three-column osteotomy, *SRS-22* Scoliosis Research Society-22 scores.

*Significant (*P* < 0.05) compared with SSM group before and after treatment.

**Significant (*P* < 0.05) compared with that before treatment in SSM and 3-COS groups.

^a^Represents SRS-22 scores at final follow-up.

## Discussion

The ASD has been traditionally treated with SSM which is still the most common surgery for ASD patients. Three-COS is commonly required for corrections of severe ASD including coronal, axial, and sagittal spinal misalignments with or without global spinal imbalance. The optimal surgery is yet to be determined with comparison between SSM and 3-COS. In this study, we compared the characteristics, surgical data, complications, and clinical outcomes of ASD patients with 3-COS and SSM. Our findings demonstrate that 3-COS can improve clinical outcomes in severe ASD patients more than SSM.

The SRS-22 outcomes questionnaire is a valid instrument for the assessment of the health-related quality-of-life of ASD patients [22,23]. In our study, 3-COS could improve the total score more significantly at final follow-up than at pre-operation; however, no significant change was found in the SSM group. This result indicates that 3-COS is more effective for correcting severe ASD than SSM. Nevertheless, compared with SSM, the function and pain scores of 3-COS groups were lower. This may be due to the severe ASD producing severe pain.

Three-COS has been performed for correction of ASD. However, the incidence of complications was high [24,25]. Daubs et al*.* [11] presented the complication rate was 37% in 46 complex ASD patients. It has been reported that infection is a major complication in ASD patients who underwent correction surgery [26]. The prevalence of infection after ASD surgery has been reported to vary between 4.7% [27] and 8% [28]. In our study, 18 patients (28.1%) with SSM and 11 patients (22.9%) with 3-COS suffered complications including infection, neurological complication, implant trouble, pseudarthrosis, and adjacent segment problem and proximal junctional kyphosis (ASP and PJK) after surgery. Although no significant difference was found between these two groups, the SSM group suffered more complications than the 3-COS. In addition to the implant trouble, other complications in SSM group were higher than 3-COS group. These results suggest that the incidence of complications in the patients with SSM is slightly higher than those patients with 3-COS.

Several studies have found that the ASP and PJK are complications in the correction surgery of ASD and may cause reoperation. Yagi et al*.* [29] reported that 32 of 157 patients had PJK complications and 10 of them (6.4%) need reoperation. DeWald and Stanley [30] considered that PJK are common in ASD surgery with a high rate of 26%. Kim et al. [31] reported that 17% of the patients in their study underwent reoperation for pseudarthrosis, and significant risk factors were long fusion, sacral involvement, and preexisting thoracic hyper-kyphosis. In our study, 63 patients need reoperation, including 39 patients (34.8%) in the 3-COS group and 24 patients (21.4%) in the SSM group. We suggest that the higher reoperation rate in the 3-COS group may be due to the complex operations of large corrections.

Many studies showed that age is a significant risk factor for complications after ASD surgery, overall complication rates were significantly higher among older patients [4,8,11,32-34]. Older patients often recovered slowly after surgery. In our study, the average age of ASD patients at the initial surgery was 52.5 years, thus, old age may be a cause of the high complication rates. Further, history of medical complications [4,9,32] and fusions to the sacrum [32-34] are also risk factors for complications in ASD patients who underwent ASD surgery. In our study, most patients suffered one or more comorbidities, and approximately 80% patients with SSM and 3-COS were fused to sacrum. In addition, radiographic parameters (sagittal imbalance, PT of 26° or more) were found to be a risk factor for complications in patients who underwent primary ASD surgery [32]. Lafage et al. [12] described that SVA, PT, and LL proportional to PI were strongly correlated with health-related quality-of-life (HRQOL) and PI/LL mismatch caused high PI, low LL, or both. With respect to the radiographic parameters in our study, the preoperative SVA, PI, PT, and PI minus LL of the patients with 3-COS were significantly higher than those with SSM. Thus, we can suggest that 3-COS might improve the clinical outcomes in correcting more severe ASD with high grade PI, PT, and PI/LL mismatch and for ASD patients who have subjected to spine surgeries more than twice before in comparison with SSM.

This study has several limitations. Firstly, the subtypes of 3-COS and SSM cannot be analyzed due to the lack of the samples. Secondly, relatively high complications were observed in both surgeries. Therefore, both surgeries should be improved further. Despite the limitations, our study still provides significance in clinical application.

## Conclusions

For severe ASD patients with high grade PI, PT, and PI/LL mismatch and who have subjected to spine surgeries more than twice before, 3-COS might be more effective than SSM in improving the clinical outcomes. However, due to the higher reoperation rate of 3-COS, SSM may be more appropriate than SSM for correcting the not serious ASD patients. Our findings will help the surgeon understand the difference between 3-COS and SSM surgeries more deeply and help patients participate in the informed decision-making regarding surgery.
